# Urbanization and the temporal patterns of social networks and group foraging behaviors

**DOI:** 10.1002/ece3.5060

**Published:** 2019-04-01

**Authors:** Teri B. Jones, Julian C. Evans, Julie Morand‐Ferron

**Affiliations:** ^1^ Department of Biology University of Ottawa Ottawa Ontario Canada; ^2^ School of Environmental Sciences University of Liverpool Liverpool UK

**Keywords:** behavioral consistency, network repeatability, social network analysis, sociality, urban ecology

## Abstract

Urbanization causes dramatic and rapid changes to natural environments, which can lead the animals inhabiting these habitats to adjust their behavioral responses. For social animals, urbanized environments may alter group social dynamics through modification of the external environment (e.g., resource distribution). This might lead to changes in how individuals associate or engage in group behaviors, which could alter the stability and characteristics of social groups. However, the potential impacts of urban habitat use, and of habitat characteristics in general, on the nature and stability of social associations remain poorly understood. Here, we quantify social networks and dynamics of group foraging behaviors of black‐capped chickadees (*N* = 82, *Poecile atricapillus*), at four urban and four rural sites weekly throughout the nonbreeding season using feeders with radio frequency identification of individual birds. Because anthropogenic food sources in urban habitats (e.g., bird feeders) provide abundant and reliable resources, we predicted that social foraging associations may be of less value in urban groups, and thus would be less consistent than in rural groups. Additionally, decreased variability of food resources in urban habitats could lead to more predictable foraging patterns (group size, foraging duration, and the distribution of foraging events) in contrast to rural habitats. Networks were found to be highly consistent through time in both urban and rural habitats. No significant difference was found in the temporal clumping of foraging events between habitats. However, as predicted, the repeatability of the clumping of foraging events in time was significantly higher in urban than rural habitats. Our results suggest that individuals living in urban areas have more consistent foraging behaviors throughout the nonbreeding season, whereas rural individuals adjust their tactics due to less predictable foraging conditions. This first examination of habitat‐related differences in the characteristics and consistency of social networks along an urbanization gradient suggests that anthropic habitat use results in subtle modifications in social foraging patterns. Future studies should examine potential implications of these differences for variation in predation risk, energy intake, and information flow.

## 1 INTRODUCTION

Habitat change through urbanization presents major environmental challenges to animals. These include shifts in disturbance levels, pollution, community composition, and resource abundance (reviewed by Garcia, Suárez‐Rodríguez, & López‐Rull, 2017). To address these environmental challenges, animals occupying urban habitats may exhibit different behavioral responses than conspecifics living in less altered rural habitats (reviewed by Miranda, 2017). For example, urban populations are often found to express more risk‐prone, more aggressive, and less neophobic behaviors than their rural counterparts (Lowry, Lill, & Wong, 2013). These differences might also lead to changes in animals’ social behaviors, with the responses of group members interacting to influence the overall group social structure (Öst, Seltmann, & Jaatinen, 2015; Tanner & Jackson, 2012). Changes in social structure have an important impact on group living species, as an individual's social associations and position within their social group can influence their fitness, for instance, by impacting reproductive success and offspring survival (Cheney, Silk, & Seyfarth, 2016; Wey & Blumstein, 2012), or through influencing access to mating opportunities (Formica et al., 2012; McDonald, 2007; Oh & Badyaev, 2010).

Although urban and rural habitats differ across a variety of features (Garcia et al., 2017; Isaksson, Rodewald, & Gil, 2018), it has been suggested that differences in the availability and distribution of resources are particularly significant to avian communities (Chace & Walsh, 2006; Tryjanowski et al., 2015). Variation in the abundance and distribution of resources has been suggested to alter the formation and behavior of animal groups (Johnson, Kays, Blackwell, & Macdonald, 2002); higher abundance of resources in urban environments may thus cause changes to the structure of social groups and stability of individual's positions within those groups. For example, if an advantage gained from group behavior is enhanced food finding due to access to social information, the value of sociality might be eroded if finding food requires little effort in urban habitats (Jones, Aplin, Devost, & Morand‐Ferron, 2017).

As urbanization is occurring at a rapid rate, it is important to consider how the behavior and stability of natural animal social groups are impacted by urban living. However, few studies have considered how social groups and behaviors may vary with differing environmental conditions (Belton, Cameron, & Dalerum, 2018; Blaszczyk, 2017; Stanley & Dunbar, 2013), and only two studies have considered how disturbances to the environment may influence the stability of networks (Formica, Wood, Cook, & Brodie, 2017; Krause et al., 2017). In this study, we provide the first assessment, to our knowledge, of variation in the characteristics and consistency of group foraging behaviors and temporal stability of individual social network position in wild urban and rural groups. We measured the social networks and foraging behaviors of black‐capped chickadees (*Poecile atricapillus*) at four urban and four rural sites during the nonbreeding season. Chickadees form flocks during the autumn, remain in resident groups on a communal home range throughout the winter, and commonly forage socially at both natural and artificial food sources (i.e., bird feeders). As food availability has been found to limit winter survival in chickadees (Desrochers, Hannon, & Nordin, 1988), social associations may be of particular importance if they can increase the ability of individuals to locate foraging patches and thus minimize the risk of starvation, through the use of social foraging tactics (Galef & Giraldeau, 2001).

We therefore predict the following: Social networks in urban habitats will be less stable than rural networks over time, as the abundance of artificial resources reduces the value of enhanced food finding via social foraging tactics. Moreover, arrivals of individuals to group foraging events in urban areas will be more spread out in time (less temporally clumped), due to groups being less cohesive. Less cohesive groups could also lead to foraging events lasting longer in urban areas, as dispersed groups may be less efficient at exploiting the resources (Maldonado‐Chaparro, Alarcón‐Nieto, Klarevas‐Irby, & Farine, 2018) and/or consisting of fewer individuals. Further, the persistence of artificial resources at urban sites could lead to longer foraging events. These patterns for foraging events will be more consistent over time in urban areas, where individuals are expected to be less affected by seasonal environmental changes (Lowry et al., 2013).

## 2 METHODS

### 2.1 Study system

Black‐capped chickadees are small (9–14 g) passerines that are year‐round residents throughout their range in most of North America (Foote et al., 2017). During the nonbreeding season (September–April), birds form social groups with strongly linear dominance hierarchies that generally range in size from eight to 12 individuals (Desrochers et al., 1988; Smith, 1991). Throughout the winter, groups maintain a local home range which has been found to vary in size from approximately 8.8 to 22.6 ha (Smith, 1991).

Birds were captured at eight study sites located in and around Ottawa, ON, Canada (45°25′N, 75°40′W), between 26 September and 9 December 2014, using mist nets and potter's traps baited with sunflower seeds. The four urban sites were located within 7 km of Ottawa's downtown core and consisted of partially forested city parks of at least 200 x 200 m in size. Residential housing, likely to contain supplemental feeders (confirmed feeders at three of the four urban sites; personal observation), was found within 90 m of each urban site. The four rural sites were located in large forested patches >15 km from Ottawa's downtown, and all contained no buildings within 300 m of the study location, and thus, we expect rural groups not to have access to supplemental feeding in their home range beyond that of our study feeder. Birds were equipped with a passive integrated transponder (PIT) tag (IB Technology, UK) to allow for automated recording of social associations, as well as a Canadian Wildlife Service‐issued aluminum band and an additional plastic color band to allow for visual identification. At each site, a sunflower seed feeder fitted with a single modified perch which contained a radio frequency identification (RFID) antenna (Priority 1 Design, Australia) recorded visits by marked individuals and was filled once per week, which allowed visible food to be present in the feeder throughout each week. Sites were located >2 km apart, which is beyond the expected range of movements for wintering chickadees, and no individual was recorded at more than one site.

### 2.2 Social networks

We recorded data from 1 November 2014 to 5 January 2015 and 29 January to 31 March 2015, and all sites had a minimum of 13 weeks of collection (range: 13–18). Social associations were determined from temporal data collected from marked individuals using Gaussian mixture models (GMMs; Psorakis, Roberts, Rezek, & Sheldon, 2012; Psorakis et al., 2015; Evans, Jones, & Morand‐Ferron, 2018). GMM detects “bursts” of increased activity, to which individual events are assigned (Farine et al., 2015; Psorakis et al., 2012, 2015). In our case, these bursts represent foraging events. Following the gambit of the group approach (Franks, Ruxton, & James, 2010; Whitehead & Dufault, 1999), individuals co‐occurring in the same foraging event were categorized as associating. To assess network consistency across the study period, we constructed a separate undirected weighed social network for each week, at each site, using the simple ratio index (SRI; Cairns & Schwager, 1987), a measure of association between individuals which ranges from 0 (pair never seen associating) to 1 (never detected apart), using the R package asnipe (Farine, 2013). Birds were excluded from analysis if they were recorded in less than five sampling weeks, as assessment of consistency requires that individuals were repeatedly sampled and a bird was determined present in a given week only when recorded greater than five separate times as very low frequency of visits suggests a lack of residency in the study site (Evans et al., 2018).

Weighted degree centrality and eigenvector centrality were calculated for each individual, in each weekly foraging network using igraph package (Csardi & Nepusz, 2006). Weighted degree centrality is an individual's number and strength of direct connections and can be seen as a general measure of how social an individual is (Wey, Blumstein, Shen, & Jordán, 2008). Eigenvector centrality is an indication of an individual's connectedness in the network defined from the principal eigenvector of the network matrices (Borgatti, 2005) and is proportional to the sum of the centralities of an individual's neighbors (Brent, 2015; Farine & Whitehead, 2015). Eigenvector centrality is an important metric of social position, especially in terms of measuring “flow” through groups, for instance, in the case of information or disease transmission (Borgatti, 2005; Brent, 2015). Highly central individuals thus have the potential to reach or influence other individuals in the network faster than individuals occupying more peripheral network positions.

### 2.3 Network consistency

We tested for consistency in overall network positions using the methods suggested by Wilson, Krause, Dingemanse, and Krause (2013), to account for the nonindependent nature of social network data. Within each site, we calculated the variance in each individual's ranked weighted degree and eigenvector centrality across all weekly networks. The sum of each individual's variance in ranks (SV) was then used as a measure indicating the overall change in network positions occurring between the weekly networks. This results in a single observed variance value (SV_O_) for each network metric at each site. Low values of SV_O_ indicate a similar relative ranking of individuals across weekly networks, whereas a high value would indicate that individuals’ network positions are less consistent. The observed values SV_O _were then compared to the sum of variance values obtained when repeating the analyses on randomized network (SV_R_). For every sampling week at each site, we generated 1,000 random networks using data‐stream permutation (Farine, 2017; Farine & Whitehead, 2015). Within a week, individuals were swapped between foraging events occurring on the same day, producing increasingly random networks. Significance was calculated as the proportion of SV_R _values from randomized versions of a network that were smaller than the SV_O_ obtained from the observed network. Networks were deemed to be significantly consistent if this proportion was less than 0.05. We also calculated the adjusted repeatability of individual's ranked network metrics, defined as the proportion of total variation explained by between‐groups variance (Nakagawa & Schielzeth, 2010), with month (as a categorical variable in order to control for possible seasonal changes in foraging behavior) and week number as fixed effects and site as a random effect. The R values of the observed networks were compared to those obtained from the randomized networks. Significance was calculated as the proportion of *R* values from randomized networks that were larger than the *R* value obtained from the real network. Individual network position was deemed to be significantly repeatable if *p* was less than 0.05. Additionally, we also carried out similar repeatability analysis to urban and rural subsets of the data separately in order to see if repeatability of individual network metrics differed between urban and rural groups. In these models, site was used as a fixed effect rather than a random effect due to the low number of replicates (*N* = 4) per habitat types (Bolker et al., 2009). Repeatabilities were considered to significantly differ between habitat types if their 84% confidence intervals did not overlap, since a lack of overlap between the group's confidence intervals is equivalent to a 95% confidence interval around the difference not including zero (Julious, 2004). The significance of these repeatabilities was also calculated via comparison to the repeatabilities calculated from randomized versions of the network. All repeatabilities were calculated using the rptR package (Stoffel, Nakagawa, & Schielzeth, 2017).

### 2.4 Quantifying social foraging behavior

**Figure A1:**
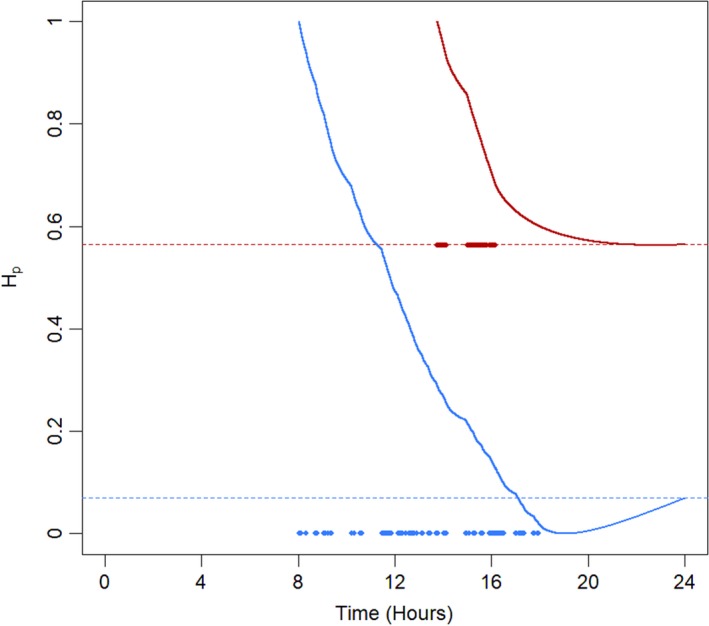
Illustration of clumpiness measure Entropy (H_p_) for two days exhibiting high clumpiness (red) and low clumpiness (blue). Points represent arrival events (first arrival of an individual in a foraging event) and dotted lines represent the final value of H_p_ for that day. The solid line shows the changing value of H_p_ as it would be measured at that time‐point. For further information see Zhang, Bradlow, & Small (2013)

In order to quantify the ways in which groups foraged at the RFID feeder, we calculated the number of individuals present, and the duration of and the “clumpiness” of group foraging events. “Clumpiness” of events can be defined as a measure of how spread out events are in time. Regularly spaced events will result in a low amount of clumpiness, whereas events which are highly clustered together in time will result in a high amount of clumpiness. We use entropy (H_p_) as our measure of clumpiness as in Zhang, Bradlow, and Small (2013). Over the course of each sampling period (a day), we calculated H_p_ as

$$H_{p}=\sum_{i=1}^{n+1}x_{i}log\left(x_{i}\right)$$

where *n *is the number of intervals between events, *x_i_* is the *i*th interval between subsequent events (in seconds), *x_1_*is the interval between the beginning of the sample period and the first event, and *x_n + 1 _*is the interval between the end of the final event and the end of the sampling period *N* (the end of the day). The resulting values of each day's *H_p_* are then rescaled between 0 and 1, where 0 is the least clumpy and 1 is the most. For an illustration of this measure, see Appendix Figure A1. We calculated the *H_p _*of each individual's initial arrival to each foraging event throughout the day; we termed this “clumpiness of arrivals,” which is a measure of the cohesiveness of foraging groups. We also calculated the *H_p_* of foraging events, measured from the arrival time of the individual that starts the foraging event to the departure time of the final individual to leave, throughout each day, which we term “clumpiness of events,” which indicates the pattern of resource use throughout the day.

### 2.5 Statistical analysis of foraging metrics

Six models were fitted to test the effect of habitat type on foraging patterns. To explore daily foraging patterns, we examined the number of foraging events per day, number of visits per day, the clumpiness of arrivals within a day, and the clumpiness of foraging events within each day. While it might be expected that a greater total number of visits per day will lead to an increase in the number of detected foraging events, the opposite may also be expected if group foraging consists of a large number of short visits per event. At the level of the foraging event, we examined the number of individuals present and the duration of foraging events, clumpiness of foraging arrivals, and clumpiness of events. As well as habitat type, we also fitted month and the number of individuals at that site (to control for any effects of having differing numbers of potential foragers), and all possible two‐way interactions as fixed effects. Site was fitted as a random effect. The models examining duration of foraging events and clumpiness metrics were fitted with a gaussian distribution, while all other models were fitted using a poisson distribution. All model assumptions were checked (i.e., absence of overdispersion and normality of residuals). All possible model combinations were fitted and a model selection procedure was performed using Akaike's information criterion (AICc; AIC corrected for small sample size, Burnham & Anderson, 2004) to evaluate predictor effect size. We then ranked models using Akaike weight (AIC_w_) as a measure of relative importance of each model parameter (Burnham & Anderson, 2004). For competing models with a ΔAICc < 2, we carried out model averaging. All statistical analyses were performed in R v3.2.3 (R core team, 2015) using package lme4 (Bates, Mächler, Bolker, & Walker, 2015) for model construction and MuMIn for model selection (Bartoń, 2016).

To calculate the adjusted repeatability of group foraging metrics for each site, we fitted overall group size, month (categorical), and day of study as fixed effects, with site as a random effect. The model examining number of individuals per foraging event, number of foraging events per day, and number of visits per day was once again fitted with a Poisson distribution. As with the repeatability analysis of network metrics, we repeated this analysis on urban and rural subsets of the data in order to test for differences in repeatability of foraging metrics for urban and rural groups. Once again, repeatabilities were considered to significantly differ between habitat types if their 84% confidence intervals did not overlap (Julious, 2004).

## 3 RESULTS

Over the recording period, a total of 155,138 visits were made to the eight network feeders by 91 birds; analysis was restricted to 82 individuals as nine individuals did not meet the minimum recording criteria and thus were excluded. Weekly networks at each site ranged from four to 15 birds.

### 3.1 Network consistency

The observed sum of variance (SV_O_) was found to be significantly lower than the sum of variance from randomized networks (SV_R_) at all eight sites when considering both an individual's weighted degree centrality and their eigenvector centrality (Table 1). Both network metrics were found to be significantly repeatable, with no significant difference in repeatability between urban and rural individuals (Table 2).

**Table 1:** Consistency of individual network position, as measured by the sum of variance (SV_O_) in ranked network metrics (weighted degree centrality and eigenvector centrality)

Site	Habitat type	Number of individuals	Weighted degree centrality		Eigenvector centrality	
			SV_O_	*p* value	SV_O_	*p* value
Aviation Parkway	Urban	10	0.39	<0.01	0.39	<0.01
Carlington Woods	Urban	11	0.57	<0.01	0.58	<0.01
Hampton Park	Urban	10	0.59	<0.01	0.59	<0.01
Pleasant Park	Urban	9	0.33	<0.01	0.29	<0.01
Bell Bushlot	Rural	11	0.49	<0.01	0.48	<0.01
South Marsh	Rural	12	0.52	<0.01	0.54	<0.01
Stony Swamp	Rural	17	0.56	<0.01	0.54	<0.01
Wolf Grove	Rural	10	0.54	<0.01	0.54	<0.01

Note

*p* values obtained by comparing the SV_O _of the real network to 1,000 randomized versions of the networks

**Table 2:** Adjusted repeatabilities of weighted degree centrality and eigenvector centrality, calculated for all groups, followed by separate values for urban or rural groups

Metric		*R*	2.5% CI	97.5% CI	*p* value
Weighted degree centrality		0.38	0.28	0.46	<0.05

	Habitat	*R*	8% CI	92% CI	*p* value
Weighted degree centrality	Urban	0.41	0.31	0.49	<0.05
Weighted degree centrality	Rural	0.39	0.3	0.48	<0.05

Metric		*R*	2.5% CI	97.5% CI	*p* value
Eigenvector centrality		0.45	0.35	0.53	<0.05

	Habitat	*R*	8% CI	92% CI	*p* value
Eigenvector centrality	Urban	0.43	0.34	0.52	<0.05
Eigenvector centrality	Rural	0.52	0.43	0.6	<0.05

Note

95% confidence intervals for all groups and 84% confidence intervals for urban or rural groups calculated via parametric bootstrapping.

*p* values calculated via comparison to the same models carried out on randomized networks

### 3.2 Social foraging behavior

**Figure 1:**
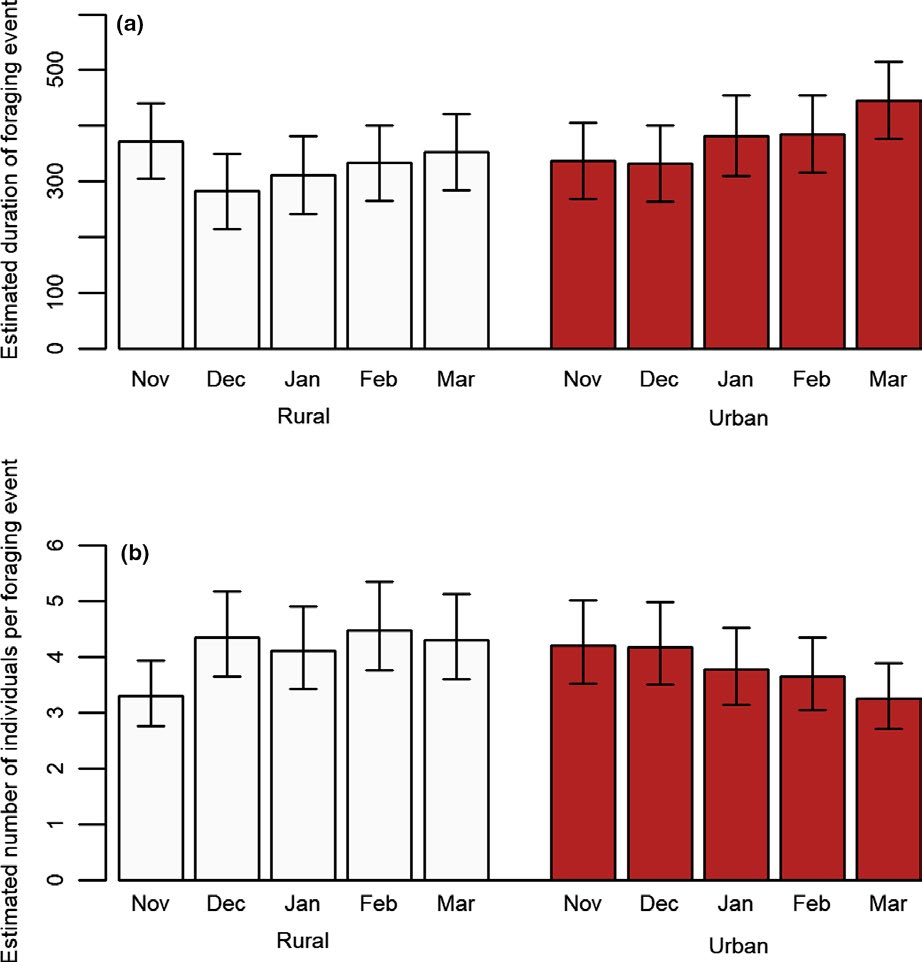
Figure showing the estimated effect of habitat type and month on (a) on the duration of foraging events (in seconds) and (b) the number of individuals in a foraging event, with 95% confidence intervals

**Figure 2:**
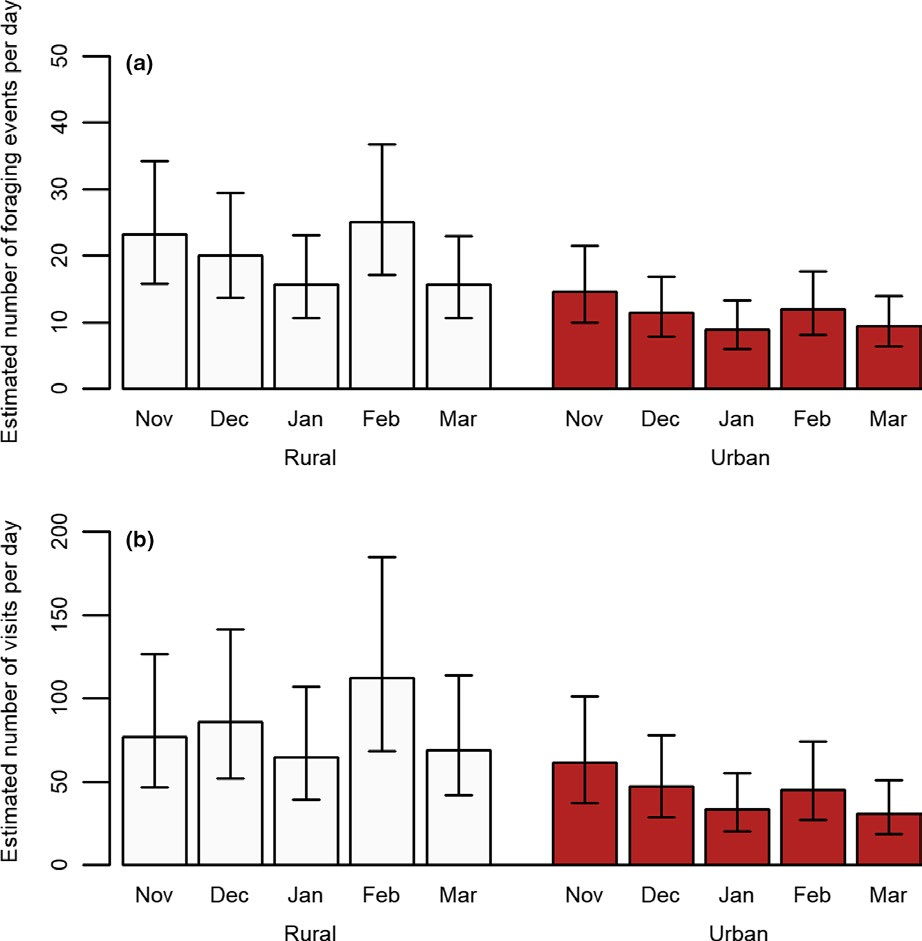
Figure showing the estimated effect of habitat type and month on (a) the number of foraging events per day and (b) total number of visits per day, with 95% confidence intervals

Model selection for the duration of foraging events found a single top model consisting of habitat type, month, and their interaction (Appendix Table A1). This model indicated the duration of foraging events in urban habitats was greater in months later in the winter compared with November (Figure 1a). Model selection for the number of individuals in a foraging event also found a single top model consisting of habitat type, month, and the interaction between them (Appendix Table A2). The model suggested that the number of individuals in foraging events in urban areas decreased slightly in the late winter months of the season in comparison with November, whereas rural groups showed no consistent pattern over time (Figure 1b). Model selection for both number of foraging events per day (Appendix Table A3) and total number of visits per day also suggested similar top models (Appendix Table A4). Here, there were no clear differences in how these metrics were altered by month, though both metrics in both habitat types peaked at February (Figure 2). We additionally conducted an analysis in which we considered date as a continuous variable (details in the appendix) to test for continuous changes in foraging over time, with similar findings (Appendix Tables A5, A6, A7, A8). For both clumpiness of arrivals and clumpiness of foraging events, the null model was found to be the single best fitting model (Appendix Tables A9 and A10), with no parameters found to have a significant effect on clumpiness metrics.

### 3.3 Repeatability of social foraging behavior

The number of individuals in a foraging event showed low repeatability overall and the repeatability of the duration of foraging events was extremely weak, with no significant difference in repeatability between habitats (Table 3). Repeatability of number of visits and number of foraging events per day was extremely weak overall and also when considering urban and rural groups separately (Table 4). Clumpiness of arrivals to foraging events was found to have low repeatability overall (Table 4). Among urban groups, the clumpiness of arrivals to foraging events was found to be moderately repeatable and significantly more repeatable than the clumpiness of arrival to foraging events in rural groups which was not found to be significantly repeatable (Table 4). Clumpiness of foraging events was moderately repeatable overall, and once again, urban groups were moderately repeatable while rural groups were not repeatable (Table 4). However, there was no significant difference between the repeatabilities due to overlapping 84% confidence intervals.

**Table 3:** Adjusted repeatabilities of number of individuals per foraging event and duration of foraging events calculated for all groups, followed by separate values for urban or rural groups

Metric		*R*	2.5% CI	97.5% CI	*p* value
Individuals in foraging event		0.11	0.03	0.2	<0.01

	Habitat	*R*	8% CI	92% CI	*p* value
Individuals in foraging event	Urban	0.15	0.02	0.21	<0.01
Individuals in foraging event	Rural	0.1	0.01	0.16	<0.01

Metric		*R*	2.5% CI	97.5% CI	*p* value
Duration of foraging events		0.06	0.02	0.13	<0.01

	Habitat	R	8% CI	92% CI	P value
Duration of foraging events	Urban	0.09	0.02	0.18	<0.01
Duration of foraging events	Rural	0.04	0.005	0.08	<0.01

Note

95% confidence intervals for all groups and 84% confidence intervals for urban or rural groups calculated via parametric bootstrapping.

*p* values calculated via least ratio tests.

**Table 4:** Adjusted repeatabilities of clumpiness of first arrivals to foraging events, clumpiness of foraging events, number of visits per day, and number of foraging events per day, calculated for all groups, followed by separate values for urban or rural groups

Metric		*R*	2.5% CI	97.5% CI	*p* value
Clumpiness of first arrivals		0.11	0.02	0.25	<0.01

	Habitat	*R*	8% CI	92% CI	*p* value
Clumpiness of first arrivals	Urban	0.33	0.03	0.56	<0.01
Clumpiness of first arrivals	Rural	0	0	0.02	0.5

Metric		*R*	2.5% CI	97.5% CI	*p *value
Clumpiness of foraging events		0.23	0.05	0.42	<0.01

	Habitat	*R*	8% CI	92% CI	*p* value
Clumpiness of foraging events	Urban	0.4	0.04	0.64	<0.01
Clumpiness of foraging events	Rural	0.05	0	0.14	0.07

Metric		*R*	2.5% CI	97.5% CI	*p* value
Number of visits		0.02	0.003	0.042	<0.01

	Habitat	*R*	8% CI	92% CI	*p* value
Number of visits	Urban	0.08	0.01	0.13	<0.01
Number of visits	Rural	0	0	0.001	1

Metric		*R*	2.5% CI	97.5% CI	*p* value
Number of foraging events		0.03	0.001	0.052	<0.01

	Habitat	*R*	8% CI	92% CI	*p* value
Number of foraging events	Urban	0.1	0.006	0.18	<0.01
Number of foraging events	Rural	0	0	0.002	1

Note

95% confidence intervals for all groups and 84% confidence intervals for urban or rural groups calculated via parametric bootstrapping.

*p* values calculated via least ratio tests.

## 4 DISCUSSION

Urbanization directly influences numerous aspects of the environment (Garcia et al., 2017), as well as the behaviors of animals that are able to persist in these altered habitats (Lowry et al., 2013; Miranda, 2017). Although studies have now documented how animals may alter their foraging behavior to cope with urban habitats and to exploit anthropogenic food sources (reviewed by Lowry et al., 2013), less attention has been paid to how urbanized habitats may influence social group dynamics and social foraging behaviors. In this study, we found subtle differences between the group foraging behavior of urban and rural groups of black‐capped chickadees. While habitat type did not appear to have a significant effect on many of our group foraging metrics, it did appear to influence how these metrics changed over the course of the nonbreeding season and the repeatability of some metrics. We also demonstrate that individual sociality and position within their network, measured as weighted degree centrality and eigenvector centrality, are equally consistent in both urban and rural groups of black‐capped chickadees throughout the nonbreeding season. Together, these results suggest that while urbanization may not lead to significant changes in group foraging behavior and stability of social groups, it may affect how individuals deal with seasonal changes in their environment.

We predicted that networks in urban areas may be less stable than those in rural areas due to decreased value of social information about resources. However, the consistency of individual position within a network was found to be significant at all sites and not significantly different between habitats, for both network metrics used. The robustness of individual position within the network being unaffected by urbanization may be due to several reasons. Individual network positions may be a function of dominance (Ficken, Witkin, & Weise, 1981; Modlmeier, Keiser, Watters, Sih, & Pruitt, 2014; Ward & Webster, 2016), meaning that changes in individual position would also require significant alterations to a group's dominance hierarchy. Previous research has shown that chickadee dominance hierarchies are strongly linear throughout the winter, until the breakup of the group (Devost, Jones, Cauchoix, Montreuil‐Spencer, & Morand‐Ferron, 2016; Smith, 1991). Dominant individuals will likely continue to enforce their position regardless of resource abundance (Ficken, Weise, & Popp, 1990; Smith, 1991), which may in turn constrain social interactions within the group. An individual's requirement to balance their foraging needs with maintaining social cohesion may be semi‐obligatory, granting them access to a territory without receiving aggression from the dominant individuals controlling that territory (Gaston, 1978). Alternatively, it is possible that enhanced food finding due to access to social information provides benefits even in more predictable urban habitats, or that winter sociality in chickadees is influenced by additional factors. Foraging in groups is expected to also provide important antipredation benefits (Ward & Webster, 2016) which may be equally valuable in urban and rural areas (Frid & Dill, 2002; Seress, Bókony, Heszberger, & Liker, 2011; Sorace & Gustin, 2009). Additionally, it is possible that breeding pairs form over the course of the nonbreeding season within winter groups (Ficken et al., 1981; Psorakis et al., 2012); thus, maintaining associations over the winter may be an important aspect of subsequent breeding success. In any case, our findings of network consistency across both habitat types indicate it is not variation in network stability that drives the habitat difference observed in our previous findings of increased social information use in rural habitats (Jones et al., 2017). This leaves open the possibility of a genuine increased tendency to use social information in less urbanized groups. An experimental approach would be necessary at this point to test this possibility.

Though habitat type was not a significant predictor of the number of individuals per foraging event or duration of foraging events, it did appear to cause some differences in how these measures changed during the nonbreeding season. The number of individuals foraging in urban foraging events gradually decreased during the study, whereas the duration of foraging events gradually increased (continuous time analysis; appendix). There were no patterns observed in the duration of foraging events in rural habitats across the seasons, but there was a small increase in number of individuals per event after November. The slight increase in number of individuals in rural areas could reflect increased familiarity with the feeder, particularly if rural groups are initially more neophobic of novel foraging opportunities (Griffin, Netto, & Peneaux, 2017); thus, after the initial introduction of the feeder in November, the rural groups might show an increased willingness to use and rely on the feeder, whereas urban individuals are likely already utilizing similar feeders. Another non‐mutually exclusive explanation is that conditions become more difficult in rural habitats in winter, while urban individuals will have access to numerous other supplementary feeding sources throughout winter (Tryjanowski et al., 2015).

Neither the clumpiness of individual arrivals to foraging events nor that of overall foraging events appeared to be affected by habitat type or seasonal effects. This suggests that cohesiveness of arrival and the general pattern of feeder visits throughout the day are not significantly affected by habitat type or seasonality. Although urban sites possessed other supplemental feeders besides ours, it is possible that the habitats we examined might not be sufficiently different to cause large‐scale changes or that group cohesion in chickadees is equally beneficial in both habitat types. As urban and rural environments differ in a number of features beyond resource abundance and distribution, it is difficult to disentangle these effects especially if they act differently in each habitat. For instance, social stability in rural groups may be more strongly influenced by the benefits of social foraging in a less predictable environment (Rafacz & Templeton, 2003), while habitat fragmentation in urban sites may induce group stability through limitations to movement (Ditchkoff, Saalfeld, & Gibson, 2006).

Within urban sites, both clumpiness metrics were more repeatable than in rural habitats, significantly so for the clumpiness of individual arrivals to foraging bouts. For both metrics, the repeatability in rural areas was found to be nonsignificant. Therefore, while urban habitats do not appear to result in the cohesion of arrivals or patterns of feeder usage differing from rural habitats, group foraging behaviors were possibly more consistent at these sites. This is consistent with higher repeatability in the urban habitats (i.e., lower within‐site variance), although it could also result from greater between‐site variance or a combination of both, due to repeatability being computed as between‐class variance over between‐ and within‐class variance (Nakagawa & Schielzeth, 2010). Higher repeatability in clumpiness of foraging arrivals and events in urban habitats could result, for instance, from urban individuals forming routines, moving in a consistent manner between predictable resources (McNamara, Houston, & Lima, 1994). Indeed, urban chickadees in our population express faster exploring personality types and higher repeatability for exploratory behavior compared with their rural counterparts (Thompson, Evans, Parsons, & Morand‐Ferron, 2018). Thus, individual consistency in behavioral responses may drive consistency in group foraging behavior. Further analyses of foraging behavior targeted at the individual level (e.g., Milligan, Reinder, Colle, & Sheldon, 2017) would however be required to explicitly examine this potential mechanism. Our repeatability results regarding group foraging behaviors combined with the model estimates suggest that chickadee group foraging behaviors tend to remain relatively stable in both environments, but more so in urban habitats. This result, in conjunction with recent work demonstrating that urban and rural mountain (*Poecile gambeli*) and black‐capped chickadees do not differ in food caching propensity (Kozlovsky, Weissgerber, & Pravosudov, 2017; Thompson & Morand‐Ferron, 2018), may indicate that overall foraging behaviors remain similar between urban and rural chickadee populations although urban individuals may exhibit less behavioral plasticity in group foraging behavior than those in more rural areas.

Here, we show that urban habitats do not appear to cause major changes in black‐capped chickadee group stability or group foraging behaviors. However, urban individuals did appear to be less affected by seasonality, remaining more consistent in their group foraging behaviors. These results indicate that despite social information about food theoretically being of less value in urban habitats, there are still advantages to cohesion and stable social groups in these habitats, likely driven by a combination of factors including not only social foraging but also predator protection and mating opportunities. Our study is the first to our knowledge to test for differences in social network characteristics between urban and rural habitats, a potentially important consideration to social animals that inhabit the expanding urban environment.

## CONFLICT OF INTERESTS

None declared.

## AUTHOR CONTRIBUTIONS

All authors devised the study. TBJ and JMF collected the data. JCE and TBJ conducted the analysis, wrote the manuscript, and contributed equally to the manuscript. All authors revised and approved of the final version of the manuscript.

This study was undertaken in accordance with the regulations of the University of Ottawa Animal Care Committee (permit #1759) and Environment Canada's bird banding office (banding permit #10854).

## Data Availability

Data used in this manuscript is archived in the Dryad Data Repository (https://doi.org/10.5061/dryad.92jr204).

## APPENDIX 1

### 1.1

**Table A1:** Summary of best fitting model of duration of foraging event

Fixed Effects	Estimate	SE	Lower confidence interval	Upper confidence interval	*p* value
**Intercept**	**337.11**	**34.7**	**270.54**	**403.66**	**<0.01**
Habitat type (Rural)	35.88	49.12	−58.31	130.1	0.49
Month (December)	−4.06	9.67	−22.9	15.01	0.67
**Month (January)**	**45.34**	**14.96**	**16.1**	**74.72**	**<0.05**
**Month (February)**	**48.51**	**9.99**	**28.96**	**68.11**	**<0.01**
**Month (March)**	**108.87**	**10.29**	**88.75**	**129.09**	**<0.01**
**Habitat type (Rural) : Month (December)**	−**85.79**	**13.4**	−**112.15**	−**59.64**	**<0.01**
**Habitat type (Rural) : Month (January)**	−**106.41**	**19.4**	−**144.5**	−**68.46**	**<0.01**
**Habitat type (Rural) : Month (February)**	−**87.70**	**13.72**	−**114.58**	−**60.81**	**<0.01**
**Habitat type (Rural) : Month (March)**	−**128.69**	**14.56**	−**157.29**	−**100.21**	**<0.01**

**Table A2:** Summary of best fitting model of number of individuals in a foraging event

Fixed Effects	Estimate	SE	Lower confidence interval	Upper confidence interval	*p* value
**Intercept**	**1.43**	**0.08**	**1.26**	**1.61**	
Habitat type (Rural)	−0.24	0.22	−0.24	−0.24	0.06
**Month (December)**	−**0.005**	**0.02**	−**0.005**	−**0.006**	**<0.05**
**Month (January)**	−**0.1**	**0.03**	−**0.1**	−**0.11**	**<0.01**
**Month (February)**	−**0.14**	**0.01**	−**0.14**	−**0.14**	**<0.01**
**Month (March)**	−**0.25**	**0.01**	−**0.25**	−**0.26**	**<0.01**
**Habitat type (Rural): Month (December)**	**0.28**	**0.03**	**0.28**	**0.28**	**<0.01**
**Habitat type (Rural): Month (January)**	**0.32**	**0.04**	**0.32**	**0.33**	**<0.01**
**Habitat type (Rural): Month (February)**	**0.45**	**0.03**	**0.45**	**0.45**	**<0.01**
**Habitat type (Rural): Month (March)**	**0.52**	**0.03**	**0.52**	**0.52**	**<0.01**

**Table A3:** Summary of best fitting model of number of foraging events per day

Fixed Effects	Estimate	SE	Lower confidence interval	Upper confidence interval	*p* value
**Intercept**	**2.68**	**0.19**	**2.3**	**3.07**	**<0.01**
Habitat type (Rural)	0.46	0.28	0.46	0.46	0.09
Month (December)	−0.24	0.04	−0.24	−0.24	**<0.01**
**Month (January)**	−**0.49**	**0.06**	−**0.48**	−**0.5**	**<0.01**
**Month (February)**	−**0.2**	**0.04**	−**0.19**	−**0.2**	**<0.01**
**Month (March)**	−**0.44**	**0.04**	−**0.44**	−**0.43**	**<0.01**
Habitat type (Rural): Month (December)	0.1	0.05	0.1	0.1	0.06
Habitat type (Rural): Month (January)	0.1	0.07	0.09	0.1	0.19
**Habitat type (Rural): Month (February)**	**0.27**	**0.05**	**0.27**	**0.28**	**<0.01**
Habitat type (Rural): Month (March)	0.04	0.06	0.04	0.04	0.47

**Table A4:** Summary of best fitting model of number of visits to feeder per day

Fixed Effects	Estimate	SE	Lower confidence interval	Upper confidence interval	*p* value
**Intercept**	**4.12**	**0.25**	**3.62**	**4.62**	**<0.01**
Habitat type (Rural)	0.23	0.36	0.22	0.23	0.53
**Month (December)**	−**0.26**	**0.02**	−**0.26**	−**0.26**	**<0.01**
**Month (January)**	−**0.6**	**0.03**	−**0.61**	−**0.61**	**<0.01**
**Month (February)**	−**0.31**	**0.02**	−**0.31**	−**0.31**	**<0.01**
**Month (March)**	−**0.68**	**0.02**	−**0.68**	−**0.68**	**<0.01**
**Habitat type (Rural): Month (December)**	**0.37**	**0.02**	**0.37**	**0.37**	**<0.01**
**Habitat type (Rural): Month (January)**	**0.44**	**0.04**	**0.44**	**0.44**	**<0.01**
**Habitat type (Rural): Month (February)**	**0.68**	**0.03**	**0.68**	**0.69**	**<0.01**
**Habitat type (Rural): Month (March)**	**0.57**	**0.03**	**0.57**	**0.57**	**<0.01**

### CONTINUOUS TIME ANALYSIS

We repeated the analysis of foraging events using time as a continuous variable. Time was modelled as the number of days since the start of the study (rescaled between 0 and 1 to aid in model fitting). The model of duration of foraging events using a continuous time variable produced similar results to the model using month as a categorical variable, with foraging events lasting longer later in winter (Table A5). The model examining the number of individuals per foraging event found a general decrease in the number of individuals in a foraging event in urban areas, but an increase in rural areas, though urban areas initially had more individuals per foraging event (Table A6). This is similar to the results of the model fitting month as a categorical variable. As with other models, the models of the number of foraging events and individual feeder visits per day also decreased later in winter (Table A7, Table A8).

**Table A5:** Summary of best fitting model of duration of foraging events, modelling time as a continuous variable

Fixed Effects	Estimate	SE	Lower confidence interval	Upper confidence interval	p value
Intercept	312.74	33.87	247.72	377.7	
Environment type (Rural)	12.20	47.84	−79.58	104.04	<0.01
Time (Days)	127.11	11.30	104.98	149.25	<0.01
Environment type (Rural): Time (Days)	−122.73	15.70	−153.5	−91.96	<0.01

**Table A6:** Summary of best fitting model of number of individuals in a foraging event modelling time as a continuous variable

Fixed Effects	Estimate	SE	Lower confidence interval	Upper confidence interval	*p* value
**Intercept**	**1.49**	**0.09**	**1.31**	**1.66**	
Environment type (Rural)	−0.18	0.12	−0.18	−0.18	0.14
**Time (Days)**	−**0.3**	**0.02**	−**0.3**	−**0.3**	**<0.01**
**Environment type (Rural): Time (Days)**	**0.54**	**0.03**	**0.54**	**0.55**	**<0.01**

**Table A7:** Summary of best fitting model of number of visits to a feeder per day, modelling time as a continuous variable

Fixed Effects	Estimate	SE	Lower confidence interval	Upper confidence interval	*p* value
**Intercept**	**4.12**	**0.25**	**3.63**	**4.62**	
Environment type (Rural)	0.24	0.36	0.24	0.24	0.53
**Time days)**	**−0.67**	**0.02**	**−0.67**	**−0.66**	**<0.01**
**Environment type (Rural): Time (Days)**	**0.80**	**0.02**	**0.80**	**0.80**	**<0.01**

**Table A8:** Summary of best fitting model of number of foraging events per day, modelling time as a continuous variable

Fixed Effects	Estimate	SE	Lower confidence interval	Upper confidence interval	*p* value
**Intercept**	**2.64**	**0.19**	**2.26**	**3.02**	
Environment type (Rural)	0.42	0.27	0.42	0.42	0.12
**Time days)**	**−0.39**	**0.04**	**−0.39**	**−0.39**	**<0.01**
**Environment type (Rural): Time (Days)**	**0.27**	**0.06**	**0.27**	**0.27**	**<0.01**

**Table A9:** Results of AICc model selection on models of clumpiness of arrival events throughout a day

Model parameters	df	LogL	AICc	∆AICc	Weight
**Null**	**3**	**−116.09**	**238.2**	**0**	**1**
Habitat + Month + group size + all 2 way interactions	18	**−**138.82	314.6	76.35	0

Models estimate the effect of urban‐rural habitats, group size and month of study on the clumpiness of foraging event arrivals (first arrival of an individual to a foraging event). Top model highlighted in bold. Full model provided for illustrative purposes

**Table A10:** Results of AICc model selection on models of clumpiness of foraging events throughout a day

Model parameters	df	LogL	AICc	∆AICc	Weight
**Null**	**3**	**129.55**	**‐253.1**	**0**	**1**
Habitat + Month + group size + all 2 way interactions	18	111.85	‐186.6	66.47	0

Models estimate the effect of urban‐rural habitats, group size and month of study on the clumpiness of foraging events (measured as the time from the first arrival to a foraging event to the final departure). Top model highlighted in bold. Full model provided for illustrative purposes
